# Toxicity Mechanism of Low Doses of NaGdF_4_:Yb^3+^,Er^3+^ Upconverting Nanoparticles in Activated Macrophage Cell Lines

**DOI:** 10.3390/biom9010014

**Published:** 2019-01-03

**Authors:** Edyta Wysokińska, Jakub Cichos, Agnieszka Kowalczyk, Mirosław Karbowiak, Leon Strządała, Artur Bednarkiewicz, Wojciech Kałas

**Affiliations:** 1 Hirszfeld Institute of Immunology and Experimental Therapy, PAS, 53-114 Wroclaw, Poland; edyta.wysokinska@iitd.pan.wroc.pl (E.W.); strzadal@iitd.pan.wroc.pl (L.S.); 2 Faculty of Chemistry, University of Wroclaw, 50-383 Wroclaw, Poland; jakub.cichos@chem.uni.wroc.pl (J.C.); miroslaw.karbowiak@chem.uni.wroc.pl (M.K.); 3 PORT Polish Center for Technology Development, 54-066 Wroclaw, Poland; Agnieszka.Kowalczyk@eitplus.pl (A.K.); Artur.Bednarkiewicz@eitplus.pl (A.B.)

**Keywords:** gadolinium, nanoparticles, upconversion, macrophages, lanthanides, fluorescent, toxicity, cytotoxicity, mitochondria, contrast agents

## Abstract

Gadolinium-doped nanoparticles (NPs) are regarded as promising luminescent probes. In this report, we studied details of toxicity mechanism of low doses of NaGdF_4_-based fluorescent nanoparticles in activated RAW264.7, J774A.1 macrophages. These cell lines were specifically sensitive to the treatment with nanoparticles. Using nanoparticles of three different sizes, but with a uniform zeta potential (about −11 mV), we observed rapid uptake of NPs by the cells, resulting in the increased lysosomal compartment and subsequent superoxide induction along with a decrease in mitochondrial potential, indicating the impairment of mitochondrial homeostasis. At the molecular level, this led to upregulation of proapoptotic Bax and downregulation of anti-apoptotic Bcl-2, which triggered the apoptosis with phosphatidylserine externalization, caspase-3 activation and DNA fragmentation. We provide a time frame of the toxicity process by presenting data from different time points. These effects were present regardless of the size of nanoparticles. Moreover, despite the stability of NaGdF_4_ nanoparticles at low pH, we identified cell acidification as an essential prerequisite of cytotoxic reaction using acidification inhibitors (NH_4_Cl or Bafilomycin A1). Therefore, approaching the evaluation of the biocompatibility of such materials, one should keep in mind that toxicity could be revealed only in specific cells. On the other hand, designing gadolinium-doped NPs with increased resistance to harsh conditions of activated macrophage phagolysosomes should prevent NP decomposition, concurrent gadolinium release, and thus the elimination of its toxicity.

## 1. Introduction

Gadolinium (Gd) is a heavy rare earth metal element with very strong paramagnetic properties, which result from a high number of unpaired f-electrons. This feature has led to the development of various gadolinium-based contrast agents [1]. Despite the relatively high biological tolerance (median lethal dose (LD_50_) of about 100–200 mg/kg), gadolinium is known to interfere with many biological processes [2,3,4]. Three decades of widespread use of gadolinium-based contrast agents increased our knowledge about Gd^3+^-related toxicity. It is believed that chemical instability of gadolinium chelates and Gd^3+^ ion dissociation are the primary causes of adverse effects of Gd-based contrast agents [3,5]. Its toxicity was demonstrated in numerous in vitro and in vivo studies, showing detrimental effects on mitochondrial function, iron transportation and calcium ion channels [6,7]. The recent boom of nanotechnology provided researchers and clinicians with new gadolinium formulations, in which the ion is embedded in the nanoparticle crystalline matrix, with a limited of release of Gd^3+^ ions. Lanthanide-doped gadolinium nanoparticles (NPs) should therefore be regarded as promising compounds with multiple potential applications, e.g., as combined luminescent, MRI (magnetic resonance imaging) or PET (positron emission tomography) probes [8,9]. This formulation offers many additional functionalities. While light penetrates tissues poorly, near infrared radiation (NIR) photoexcited upconverting NaGdF_4_ photoluminescent nanoparticles have been recognized as promising luminescent labels and biosensors [10,11,12,13,14]. Moreover, gadolinium-based nanoparticles have a strong magneto-caloric effect, which can make them suitable as cancer therapeutics [15]. Additionally, NPs can be targeted to specific tissues by biofunctionalization with antibodies or signaling peptides [16,17].

Biological studies often lack a deep understanding of biosafety and functional interaction of such nanoparticles with living cells, because they are mostly focused on demonstrating the functionality of newly developed nanoparticles in biological systems [18,19]. Thus, very few mechanistic studies have been performed. To date, Liu et al. demonstrated the toxic effect of free lanthanide ions on isolated mitochondria. They used concentrations as high as 100 µM (corresponding to about 30 µg/mL of Gd^3+^) in order to observe a similar effect on intact cells [20]. The general mechanism of action of nanoparticles containing heavy metals has been proposed in Refs. [21,22]. It was demonstrated on a wide panel of cell lines that toxicity of heavy metal-based NPs was related to their instability at low pH, probably in the lysosomal compartment of the cell. Then the question arises to what extent those studies are pertinent to crystalline NaGdF_4_ NPs, as each nanocompound has its own chemical properties that can increase or decrease toxicity. In this regard, gadolinium element has an encouragingly high LD_50_ value and currently, gadolinium and other lanthanide NPs are regarded as safe, as demonstrated in many studies (for review see [18]). We selected a model of activated macrophages for this study. The advantage of this model is that these cell lines represent activated macrophages that actively take up nanomaterials from the media, mimicking the behavior of macrophages and other immune cells residing in tissues that clear other tissues from foreign elements. One should keep in mind that conditions in macrophage’s lysosome differ greatly from those in other somatic cells [23]. At the cellular level, mitochondrial dysfunction was detected already for Gd^3+^ concentrations as low as millions of atoms per cell [24], but these experiments were performed on isolated mitochondria. Whole-cell studies that showed toxic effects of gadolinium NPs required concentrations as high as approx. 10–100 µg/mL [25,26]. Therefore, biological inertness should be based on the lack of a specific way of entering the cell. Additionally, some cells can deposit unwanted materials with no detrimental effect on their functions. For instance, gadolinium was detected *postmortem* in the brain tissue of a great number of people without clinical signs of its toxicity [27,28].

Despite the growing interest in lanthanide-doped gadolinium nanoparticles and a wide range of new formulations, a detailed data analysis of their biological activities is still scarce. We have found in previous studies that lanthanide-doped NaGdF_4_ nanoparticles can be toxic to the RAW264.7 macrophage cell line, and demonstrated that this effect could be avoided by coating nanoparticles with Poly(ethylene glycol) (PEG2000) or silica shell [29]. Unfortunately, the coating greatly affects the size and photochemical properties of nanoparticles, and additionally, it can also be stripped within the cell [30]. The toxicity of uncoated and relatively stable NaGdF_4_ nanoparticles in macrophage cells prompted us to study this phenomenon in more detail. Here, we inquire about the molecular events underlying the toxic effect of NaGdF_4_ observed against macrophage cell lines, RAW264.7 and J774A.1. We also asked the question to what extent the formulation of Gd as a nanoparticle impacts its toxicity. We are convinced that understanding the cellular fate of gadolinium-based nanoparticles is of great importance to continue the work with these promising materials and it will help to develop more biologically inert and safe compounds desirable in imaging and cancer therapeutics.

## 2. Materials and Methods

### 2.1. Lanthanide Nanocrystal Synthesis and Characterization

NaGdF_4_:Yb^3+^, Er^3+^ nanoparticles with a diameter of about 4 nm (S1) were synthesized using modified procedure described in [31]. Briefly, to the three neck round bottom flask 1.6 mL, 0.36 mL and 0.04 mL of 0.2 M methanolic solutions of Gd^3+^, Yb^3+^ and Er^3+^ acetates were added, respectively. Afterwards 10 mL of oleic acid and 10 mL of octadecene were added. Afterwards the flask was equipped with thermometer and with vacuum and Ar adapters. Next, the flask was heated under reduced pressure to 50 °C for 30 min. and then to 150 °C for 60 min. to remove methanol and water. The flask content was continuously stirred and the flask was refilled with Ar every 10 min. Afterwards solution was cooled down to room temperature (RT) and freshly prepared methanolic solutions of NH_4_F (5 mL, 0.4 M) and NaOH (1 mL, 1 M) were mixed and immediately added to the flask. The solution was mixed for 15 min followed by evaporation of methanol at 50 °C under reduced pressure. Afterwards the solution was heated to 315 °C (25 °C/min) under Ar atmosphere and kept at this temperature for 45 min. After thus time the solution was cooled down to RT and nanoparticles were precipitated with ethanol and centrifuged, followed by purification by means of dissolution in minimal amount of hexane, precipitation with ethanol and centrifugation (repeated 4 times). The purified nanoparticles were suspended in hexane.

Nanoparticles of 16 nm (S2) were synthesized using the procedure described in [32]. Briefly, to the three neck round bottom flask 1496 mg of gadolinium trifluoroacetate trihydrate, 346 mg of ytterbium trifluoroacetate trihydrate, 38 mg of erbium trifluoroacetate trihydrate and 840 mg of anhydrous sodium trifluoroacetate were added. Afterwards 15 mL of oleic acid and 15 mL of octadecene were added. The flask was equipped with thermometer and with vacuum and Ar connectors The mixture was heated to 110 °C and degassed under vacuum for 1 h at 110–130 °C with periodic Ar refills. After this period Ar was bubbled through the solution and the flask was placed in salt bath (KNO_3_ and NaNO_3_, 1:1, *w:w*) preheated to 350 °C and kept in it for 25 min. Afterwards the mixture was cooled down to RT and nanoparticles were precipitated with ethanol and centrifuged, followed by purification by means of dissolution in minimal amount of hexane, precipitation with ethanol and centrifugation (repeated 4 times). The purified nanoparticles were suspended in hexane.

The biggest particles (S3) were synthesized using method described previously [33]. Briefly 1600 mg of Gd_2_O_3_, 391 mg of Yb_2_O_3_ and 42 mg Er_2_O_3_ were placed in beaker and moisturized with DI water. 15 mL of concentrated (38%) hydrochloric acid was added to the beaker and the mixture was heated to 100 °C. After the solution became transparent beakers’ contents were evaporated to dryness (70–80 °C). As prepared lanthanide chlorides were dissolved in 45 mL of distilled water, placed in plastic beaker followed by addition of 5 mL of concentrated (40%) hydrofluoric acid. After 15 min. of stirring, the suspension was centrifuged and the precipitate was washed three times with distilled water and placed in a Teflon bottle. Next, 50 mL of distilled water and 2 g of NaF were added to the bottle. The bottle was placed in the oil bath and heated to 105 °C for 30 min. Then the bottle was capped (a thread was wrapped with a Teflon tape for tightening) and stirred in these conditions for 24 h. White precipitate was centrifuged, washed three times with DI water and air-dried at 70 °C.

Oleic acid molecules were removed from the surface of S1 and S2 nanoparticles using modified procedure described in [34,35]. Briefly, 5 mL of nanoparticles solution in hexane was placed in the falcon flask and 5 mL of 0.05 M NOBF_4_ solution in dichloromethane and dimethylformamide (25:2, *v:v*) was added. After 5 min of vortexing white precipitate was centrifuged, washed three times with the mixture of dichloromethane and dimethylformamide (25:2), two times with ethanol and finally two times with water. Afterwards nanoparticles were suspended in water.

The composition and phase purity of nanoparticles (hexagonal phase) were determined using X-ray powder diffraction and energy dispersive spectroscopy. Particle size and zeta potential were measured on Zetasizer Nano-ZS (Malvern Instruments, Malvern, UK). Transmission electron microscopy images were captured on FEI Tecnai G^2^ 20 X-TWIN equipped with an energy dispersive spectroscopy (EDS) detector (EDAX, Mahwah, NJ, USA). All transmission electron microscopy (TEM) images were taken at 200 kV on carbon covered copper grids. Scanning electron microscopy was carried out using a Hitachi S-3400N microscope equipped with a Thermo Scientific Ultra Dry EDS detector. The corrected emission and excitation spectra were recorded on an Edinburgh Instruments FLSP 920 spectrofluorimeter. The emission decay curves were recorded with the same settings using a 60 W μs flashlamp.

For stock preparation, NaGdF_4_:Yb^3+^,Er^3+^ NPs were weighed and re-suspended in sterile water. NPs were sonicated (Sonic 0.5 ultrasonic bath, Polsonic, Warszawa, Poland) in water for 20 min (peak/period) before treatment to avoid aggregation.

### 2.2. Cell Culture and Materials

RAW264.7 and J774A.1 (mouse monocyte/macrophage cell line) cells were cultured in high-glucose Dulbecco Modified Eagle’s Medium (DMEM; IITD, Wroclaw, Poland) supplemented with 10% fetal bovine serum (FBS; Gibco, Thermo Fisher Scientific, Waltham, MA, USA), glutamate, HEPES, sodium pyruvate and Antibiotic and Antimycotic Solution (Sigma-Aldrich, St. Louis, MO, USA). Culture plates and flasks were purchased from Corning Incorporated (Tewksbury, MA, USA) and Nunc Lab-Tek II Chamber Slides were purchased from Thermo Scientific (Rockford, IL, USA). Cells were grown at 37 °C, in 5% CO_2_ and 95% humidity (NuAire, Plymouth, MN, USA). To ensure subconfluent conditions at the end of experiment, cells were seeded at densities of 10–15 × 10^3^ cells/0.1 mL (0.32 cm^2^) (cell viability assay), 22 × 10^3^–25 × 10^4^ cells/0.3 mL (0.7 cm^2^) (microscopic images), 15–16 × 10^4^ cells/0.5 mL (1.9 cm^2^) (flow cytometry), 4.8 × 10^5^ cells/1.5 mL (9.5 cm^2^) (Western blotting) and allowed to stabilize for 24 h. Trypsin-EDTA solution (IITD) was used to collect the cells. In the experiments with acidification inhibitors, cells were pre-incubated 2 h with Bafilomycin A1 or ammonium chloride NH_4_Cl (Sigma-Aldrich) before NaGdF_4_:Yb^3+^:Er^3+^ NPs exposure. Flow cytometry data were analyzed using Flowing Software 2.

For stock preparation, NaGdF_4_:Yb^3+^,Er^3+^ NPs were weighed and re-suspended in sterile water. NPs were sonicated (Sonic 0.5 ultrasonic bath, Polsonic, Warszawa, Poland) in water for 20 min before treatment to avoid aggregation. Next, NPs were diluted in culture medium, sonicated and subsequently added to cell cultures.

### 2.3. Viability Assay

Cytotoxicity of NPs was determined using the CellTiter 96 AQueousOne Solution Cell Proliferation Assay (Promega, Madison, WI, USA). Cells were treated for 48 h with the indicated concentration of NaGdF_4_:Yb^3+^,Er^3+^ NPs. Then, cells were incubated with MTS reagent (3-(4,5-dimethylthiazol-2-yl)-5-(3-carboxymethoxyphenyl)-2-(4-sulfophenyl)-2H-tetrazolium, inner salt) The values of 490-nm absorbance, corresponding to the number of metabolically active cells, were measured on a VallacVictor2 plate reader (Perkin Eliner, Waltham, MA, USA). Cell viability was calculated as a % of control (100%). Each treatment within a single experiment was performed in triplicate. In order to assess the half maximal inhibitory concentration (IC_50_) cells were treated with at least 5 increasing concentrations of NPs ranging from 0.1–50 μg/mL. The IC_50_ value was calculated accordingly to the trend curve formula.

### 2.4. Apoptosis Assays

Cells were collected after a 24-h exposure to NaGdF_4_:Yb^3+^,Er^3+^. Externalization of phosphatidylserine was detected using the Annexin V-APC apoptotic detection kit (BD Bioscience, BD Pharmingen™) according to the manufacturer’s recommendations. Apoptosis was quantified as a percentage of AnnexinV+/PI− and AnnexinV+/PI+ cells. For DNA fragmentation evaluation, cells were fixed with 70% ethanol (POCh, Gliwice, Poland) and subsequently stained with propidium iodide (50 μg/mL) and RNAse (0.02 mg/mL) (Sigma-Aldrich). Flow cytometric analysis of caspase 3 and 7 activation was detected by the CellEvent™ Caspase-3/7 Green Detection Reagent (MolecularProbes, Life Technologies). The attached cells were collected and stained according to the manufacturer’s recommendations after 24-h exposure to 1 µg/mL NaGdF_4_:Yb^3+^,Er^3+^. Cell suspensions were analyzed on FACSCalibur (Becton-Dickinson, Franklin Lakes, NJ, USA).

### 2.5. Mitochondrial Content Assay

The effect of NaGdF_4_:Yb^3+^,Er^3+^ NPs on the number of mitochondria was assessed by flow cytometry using the MitoTracker^®^ probe (Molecular Probes, Thermo Fisher Scientific) according to the manufacturer’s recommendations. After 18 h exposure to 1 μg/mL NaGdF_4_:Yb^3+^,Er^3+^, cells were stained with the MitoTracker probe in phenol red-free DMEM, washed and subjected to analysis.

### 2.6. Cell Acidification

The effect of NaGdF_4_:Yb^3+^,Er^3+^ NPs on cell acidification was assessed by the LysoTracker^®^ Green DND-26 probe (Molecular Probes, Thermo Fisher Scientific) according to the manufacturer’s recommendations. After 2 h of exposure to NH_4_Cl (5 mM) or Bafilomycin A1 (50 nM) and/or 1–2.5 μg/mL NaGdF_4_:Yb^3+^,Er^3+^ (5 h, 18 h, 24 h), cells were stained with the LysoTracker probe in phenol red-free DMEM, washed and subjected to cytometric analysis.

### 2.7. Superoxide Production

Superoxide production by mitochondria was measured by a MitoSOX™ Red mitochondrial superoxide indicator (Molecular Probes, Thermo Fisher Scientific) according to the manufacturer’s recommendations. Attached cells were collected after a 5-h exposure to 1 μg/mL NaGdF_4_:Yb^3+^,Er^3+^ and stained with the MitoSOX™ probe. Then, cells were washed and analyzed by flow cytometry.

### 2.8. Western Blotting

Whole cell lysates were prepared using cold RIPA buffer supplemented with Sigma*FAST* Protease Inhibitor Cocktail (Sigma-Aldrich). The cell lysates were then sonicated using a Sonopuls HD 2070 ultrasonic homogenizer (Bandelin, Berlin, Germany). Protein concentration was determined by the Pierce BCA Protein Assay Kit (Thermo Fisher Scientific). Protein lysates were separated by SDS-PAGE using 10 and 12% resolving gels and transferred (semi-dry) to a PVDF membrane (0.45 μm pore size; Merck Millipore, Burlington, MA, USA). Membranes were blocked with 1% casein (Sigma-Aldrich) for an hour at RT, washed with TBST (20 mM Tris, 150 mM NaCl, 0.1% Tween 20 (BioShop Canada, Burlington, NO, Canada)) and subsequently incubated with primary antibody overnight at 4 °C. After probing with HRP-conjugated secondary antibody for 1 h at RT, proteins of interest were detected using SuperSignal West Dura Extended Duration Substrate (Thermo Fisher Scientific). The following antibodies were used in this study: anti-Bcl-2 (0.1 µg/mL, #sc-7382; Santa Cruz Biotechnology, Dallas, TX, USA), anti-Bax (0.1 µg/mL, sc-6236, Santa Cruz Biotechnology), anti-LC3B (1 µg/mL, #ab51520, Santa Cruz Biotechnology), anti-actin/HRP (1:2000, #sc-1615; Santa Cruz Biotechnology), anti-mouse/HRP (1:2500, #P0447; Dako, Agilent Technologies, Santa Clara, CA, USA), anti-rabbit/HRP (1:2000–3000, #P0048; Dako, Agilent Technologies).

### 2.9. Microscopic Images

After 1-h exposure to NPs, the cells were stained with 20 nM PureBlu Hoechst 33342 in DMEM (BioRad, Hercules CA, USA) for 5 min at 37°C and washed thrice with PBS (2.5% FBS). Cells were then fixed with 4% paraformaldehyde/PBS (Sigma-Aldrich) for 30 min at 37 °C and washed. SEM images were obtained using a Hitachi S-3400N scanning electron microscope equipped with a Thermo Scientific UltraDry EDS detector. SEM images are provided with a description of the measurement conditions. Microscopic images were taken using an AxioObserverZ1 inverted fluorescence wide-field microscope (Carl Zeiss, Oberkochen, Germany) with an LD40×/0.4 Korr Ph2 objective. A BF Condenser (NA = 0.4) was used to measure bright field images, while the upconverted emission of NaGdF_4_:Yb^3+^,Er^3+^ was captured using a 975 nm laser diode (Spectra-Laser, Opole, Poland) excitation with a customized optical setup. Filter cube was composed of FF750-SDi02 dichroic and FF01-945 emission filter to cut the 976-nm excitation and transmit visible radiation. CCD AxioCam MRc5 (Carl Zeiss) and EMCCD Rolera EM-C2 (QImaging, Surrey, BC, Canada) as well as ZEN2011 (Carl Zeiss) software were used to document images.

## 3. Results

### 3.1. Physical Properties and Characterization of Luminescent Yb^3+^,Er^3+^ Co-Doped NaGdF_4_ Nanoparticles

**Figure 1:**
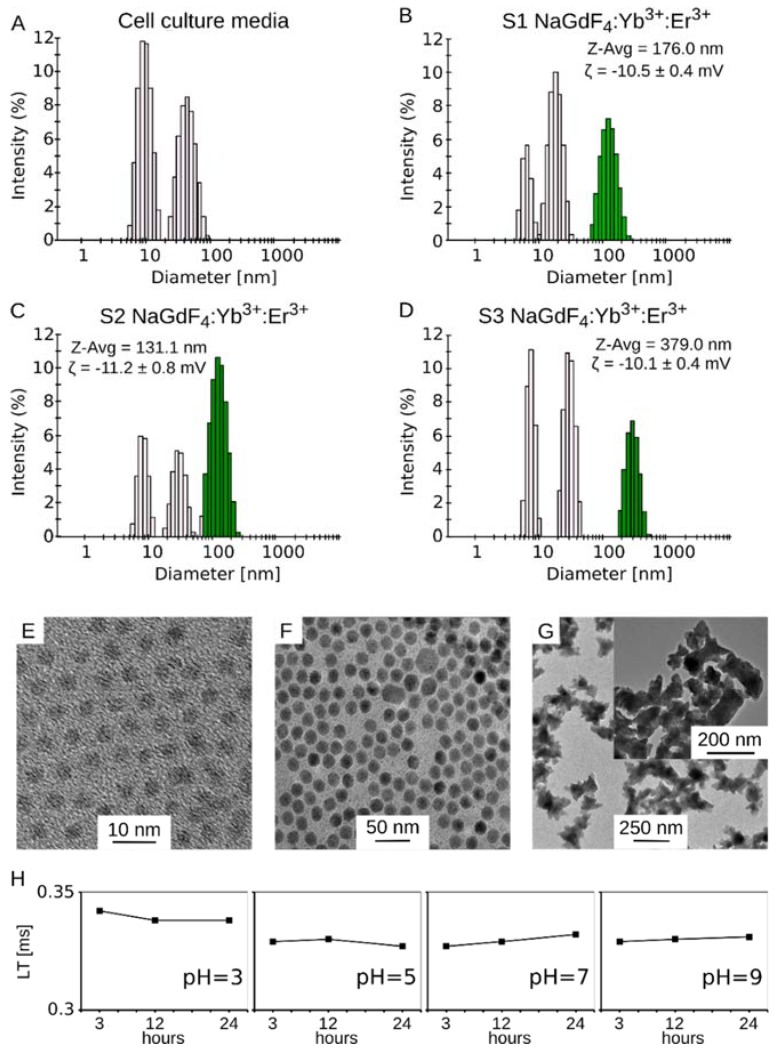
The physical characteristic of fluorescent NaGdF_4_ doped with Er^3+^ and Yb^3+^. Dynamic light scattering (DLS) histograms of (**A**) cell culture media; (**B**) small S1; (**C**) medium S2 and (**D**) large S3 NaGdF_4_ nanoparticles (NPs). Inlets: DLS—the averages of hydrodynamic size (Z-Avg) and zeta potential (ζ); (**E**–**G**) Representative transmission electron micrographs; (**H**) decay time (LT, excitation of Er^3+^ ions, λexc = 377 nm, λem = 660 nm) for S2 NPs incubated with PBS buffers with 3, 5, 7 and 9 pH value at 37 °C measured at different time points.

In this study, we have used NaGdF_4_:Yb^3+^,Er^3+^ nanoparticles with a fairly uniform zeta potential ranging from −10.1 mV to −11.2 mV (Figure 1B–D), but with different sizes (TEM): sample S1 was composed of the smallest nanoparticles with a diameter of 3.5 ± 0.4 nm (Figure 1E), sample S2 contained medium sized NPs of 16.6 ± 1.5 nm (Figure 1F), and sample S3 contained NPs with a diameter of 249 ± 59 nm (Figure 1G). S1 and S2 NPs were spherical, while S3 NPs were irregular in shape (Figure 1G inset, Supplementary Data S1). Residual oleate capping ligands on S1 and S2 NPs were removed by treatment with NOBF_4_ to ensure uniform chemical surface properties of all studied samples. These properties were measured in cell culture media supplemented with 10% FBS, because cell culture media components may greatly affect the hydrodynamic particle size. The Figure 1A shows histograms of cell culture media supplemented with fetal bovine serum. The observed peaks can be attributed to particles present in the FBS. In all cases, the third peak, which is the largest one, is a nanoparticle-related peak (marked in green in Figure 1B–D) and can be easily distinguished from FBS peaks. The average hydrodynamic sizes of small, medium and large nanoparticles were 176 nm, 131 nm and 379 nm, respectively.

A weak chemical stability of nanoparticles at low pH was implicated in some proposed toxicity mechanisms [13]. Therefore, we measured emission decay times for a series of samples at pH values equal to 3, 5, 7 and 9 (adjusted with NaOH or HCl to investigate the stability of Yb^3+^,Er^3+^ co-doped NaGdF_4_ in PBS buffer). Nanoparticles at a concentration of 3.0 mg/mL were incubated in these buffered solutions for 3, 12 and 24 h at 37 °C. Afterwards, emission decays (LT) of the ^4^F_9/2_ →^4^I_15/2_ transition were measured by monitoring the emission at 660 nm under direct excitation of Er^3+^ ions at 377 nm. Figure 1H shows that the decay time is practically independent of the pH value. Moreover, the determined decay times were very similar to the decay time for water dispersion of nanoparticles stored for 24 h. In addition, we measured emission spectra under 980 nm laser excitation to compare the green (the ^4^S_3/2_, ^2^H_11/2_ →^4^I_15/2_ transition) to red (the ^4^F_9/2_ →^4^I_15/2_ transition) intensity emission ratio (G/R ratio) of each sample. It was shown that changes in this ratio were related to depopulation of excited states of Er^3+^ due to the interaction of nanoparticles with molecules on their surface [26,27]. Analogously, changes in the G/R ratio should be observed in the case of any surface etching. However, we did not detect any changes of the G/R ratio in our experiment. These results have proved that Yb^3+^,Er^3+^ co-doped NaGdF_4_ are stable and do not undergo any decomposition during storage in PBS buffer at the 3-9 pH range. The chemical composition of each studied sample was the same as is confirmed by EDS and is given by the formula NaGd_0.80_Yb_0.18_Er_0.02_F_4_. Therefore, there is 125.8 g of Gd^3+^ in each 259.29 g of nanoparticles (0.485 g/g).

### 3.2. NaGdF_4_ Nanoparticles are Toxic Regardless of the Dopant

**Figure 2:**
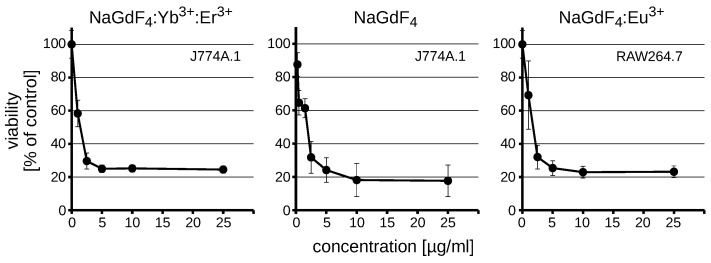
Viability of RAW264.7 or J774A.1 after a 48-h treatment with NaGdF_4_:Yb;Er (S2), NaGdF_4_ or NaGdF_4_:Eu NPs. The average ± SD are shown (*n* = 3).

We have shown in our previous studies [29] that lanthanide-doped NaGdF_4_ nanocrystals were toxic to RAW264.7 macrophages and to a lesser extend to NIH3T3 fibroblasts. The present study was complemented by the use of a similar macrophage cell line—J774A.1. The cytotoxicity of NaGdF_4_ NPs in both cell lines was assessed using the MTS assay, measuring the activity of mitochondrial dehydrogenase and marked as viability of the cell culture. This general parameter conglomerates cell death, proliferation and metabolic activity of the cells. We determined the cytotoxicity of previously used Yb^3+^,Er^3+^ co-doped NaGdF_4_ NPs along with similarly composed NPs doped with europium (Eu^3+^) ions to exclude the effect of doping element(s) on the tested toxic effect. The observed loss of viability induced by both NaGdF_4_ NPs was very similar (Figure 2, Table 1). Next, we verified whether the doping itself may influence the toxicity. We found that non-doped NaGdF_4_ NPs also exhibited similar cytotoxicity (IC_50_ = 1.13 µg/mL). These results suggest that nanoparticles are toxic regardless of the presence of the dopant and its type. Since the presence of the dopant has negligible effect on cytotoxic properties of NaGdF_4_ nanoparticles, Yb^3+^ and Er^3+^ co-doped NaGdF_4_ were selected for the current study for practical reasons related to the most suitable photoluminescence properties. Although the selected NPs are highly luminescent, their luminescence does not interfere with standard fluorescent probes used in the study (Figure S6, Supplementary Data).

The cells were treated for 48 h with increasing concentrations of NaGdF_4_:Yb^3+^,Er^3+^ NPs (0.25–10 µg/mL) of different sizes to establish the influence of NaGdF_4_ NPs on the viability of both RAW264.7 and J774A.1 lines. The observed viability loss was concentration-dependent in both cases. We also observed that S1 NPs were the most toxic, with IC_50_ less than 1 µg/mL (Table 1). The IC_50_ for S2 NPs was about 1.5 µg/mL, while the IC_50_ parameter for S3 NPs varied from 1.58 µg/mL in RAW264.7 to 4.04 µg/mL in J774A.1 cells.

**Figure 3:**
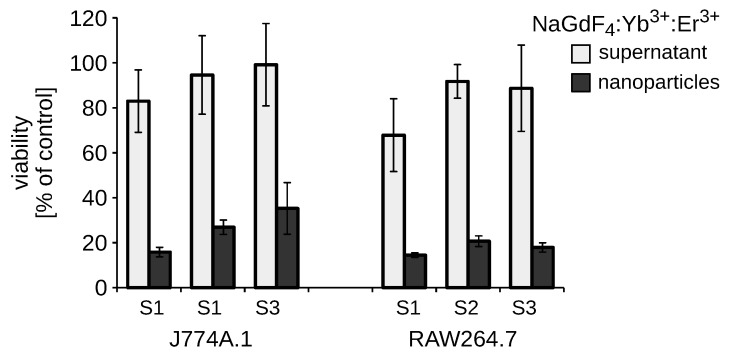
Comparison of supernatant-induced and NP-induced cytotoxicity. Viability of RAW264.7 and J774A.1 cells was assayed after a 48-h treatment with 5 µg/mL NaGdF_4_-based NPs. The means ± SD are shown (*n* = 3).

In addition, we assessed the cytotoxicity of NaGdF_4_ NPs and their supernatants separated by centrifugation to exclude the toxicity from soluble synthesis remnants (Figure 3).

### 3.3. Internalization of NaGdF_4_ Nanoparticles is Accompanied by Enlargenemt of Lysosomal Compartment and Mitochondrial Homeostasis Disruption

**Figure 4:**
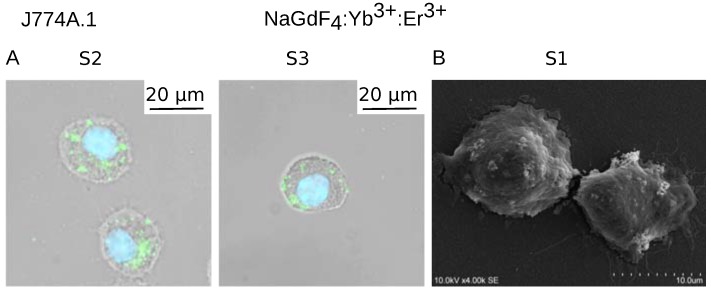
(**A**) Bright field microscopy combined with fluorescence microscopy (λ_ex_ = 975 nm; λ_em_ = 945 nm) showing interactions of S2 and S3 NaGdF_4_ NPs (5 µg/mL) with J774A.1 cells after a 1-h treatment; (**B**) SEM image of S1 NaGdF_4_ NPs.

Next, we took advantage of the luminescent properties of Yb^3+^,Er^3+^-doped NaGdF_4_ NPs and studied their interaction with the cells. Large cytoplasmic and cell membrane deposits of S2 NPs could be observed already after 1 h of treatment (Figure 4). Despite their larger size, S3 nanoparticle deposits were much less abundant, but still easy to distinguish. Most of the luminescent nanoparticles were located in both cases close to the cellular membrane. Similar results were obtained using scanning electron microscopy (SEM) (Figure S2A,B, Supplementary Data), which confirmed the presence of NPs within the cells. Emission of the smallest NPs was too weak to be observed using fluorescence microscopy, therefore, we used SEM to visualize interactions of S1 NPs with the cells. Similarly as for S2 and S3, S1 NPs could be observed close to the surface of the cells (Figure S2C). Higher microscope magnification allowed us to observe NPs within the cells.

**Figure 5:**
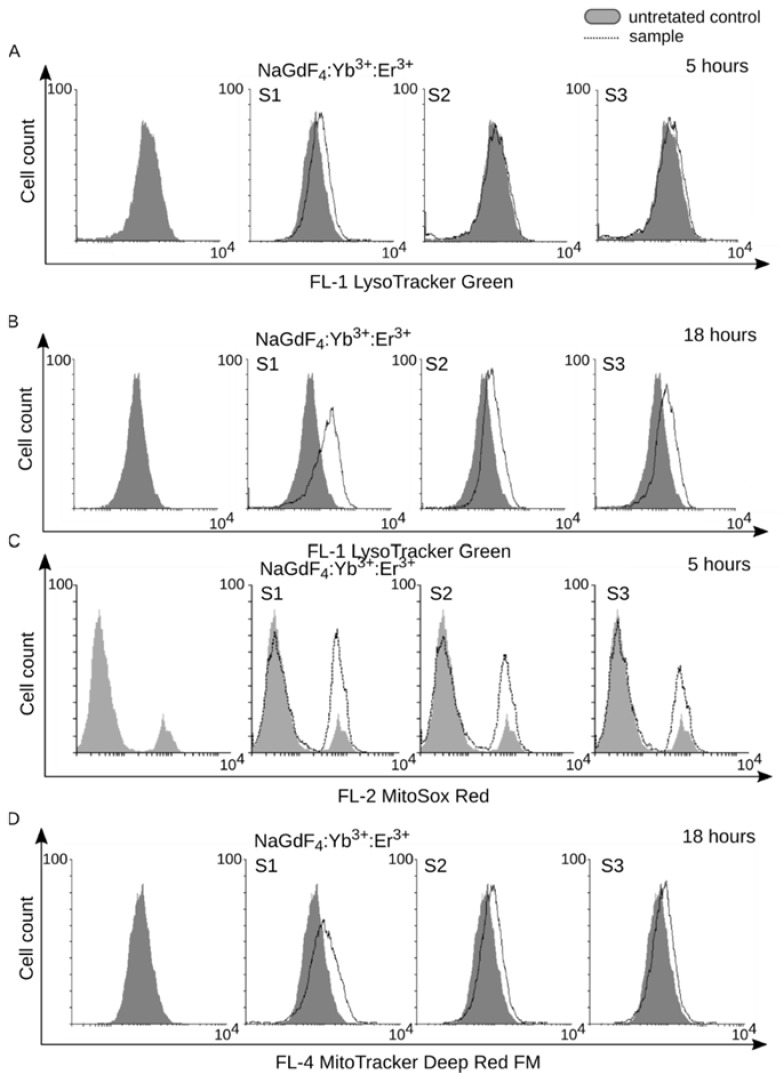
(**A**,**B**) Lysotracker analysis of cells treated with NaGdF_4_:Yb^3+^,Er^3+^ NPs (1 µg/mL) after 5 h and 18 h. A shift to the right indicates an increase of cell acidic compartment; (**C**) Induction of superoxides after a 5-h treatment with NPs detected by staining with the MitoSOX probe; (**D**) Increase of mitochondrial compartment; staining with the Mitotracker probe after an 18-h treatment with NPs. Representative histograms are shown (*n* = 3).

Both RAW264.7 and J774A.1 cell lines represent activated professional phagocytic cells. In this case, the phagocytized material is directed to phagosomes and later, after fusion with acidic lysosome, digested in phagolysosomes. These processes can lead to the enlargement of the lysosomal compartment and overall acidity of the cells, which can be measured using the Lysotracker Green fluorescent probe. We found that a 5-h treatment with NaGdF_4_ nanoparticles resulted in an increased staining of cells with the aforementioned probe (Figure 5A). The shift was most prominent for S1 NPs and very slight after treatment with larger NPs. Extending the time of treatment to 18 h resulted in a substantial increase of lysosomal compartment regardless of the size of NPs (Figure 5B). This suggests that the rate, rather than occurrence of the cytotoxic process may be size-dependent.

**Figure 6:**
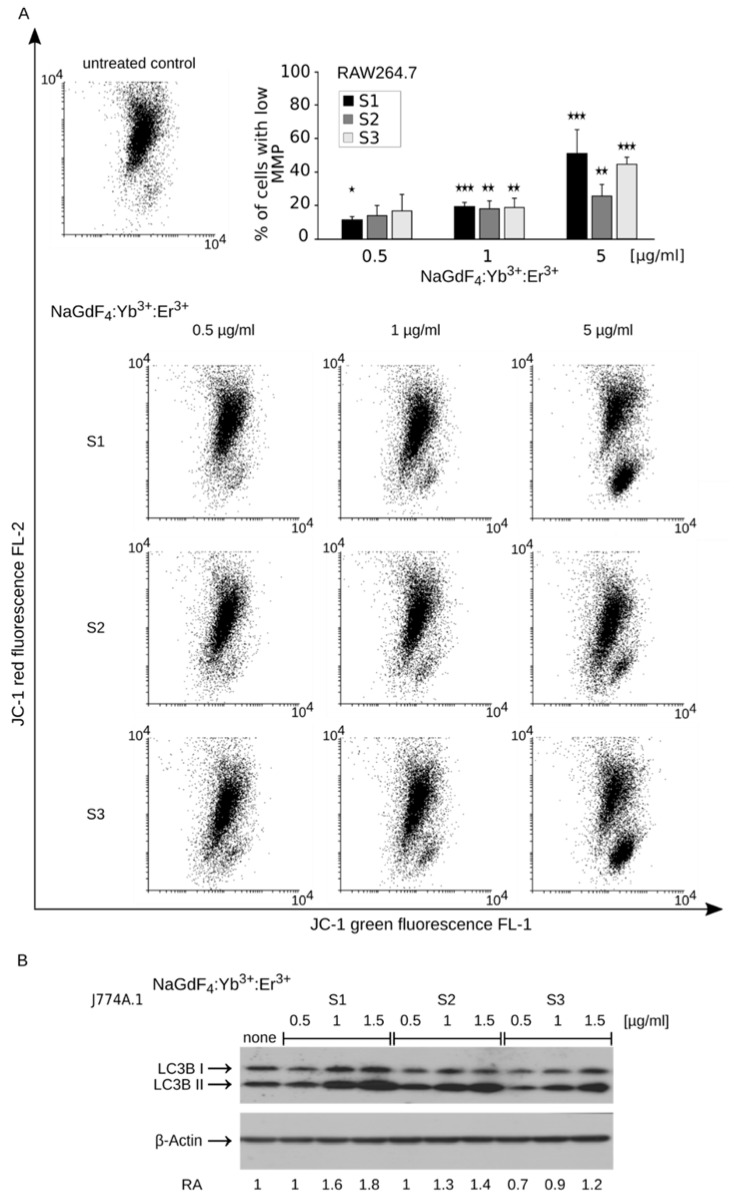
(**A**) The dot-plot analysis of the mitochondrial potential (MMP) measured using the JC-1 mitochondrial probe in RAW264.7 cells. Representative dot plots of RAW264.7 cells treated with NPs for 24 h. Bar graph shows numerical values (*n* = 3). The average percentage of cells with lowered mitochondrial ± SD are shown. * *p* < 0.05; ** *p* < 0.01; *** *p* < 0.001. All comparisons were performed with respect to the control; (**B**) Western blot analysis of J774A.1 cells treated with indicated concentrations of NaGdF_4_-based NPs for 24 h. Increased expression of the LC3B-II form can be observed as a result of treatment with NPs of all sizes. LC3B-II protein expression normalized in relation to β-Actin (RA), (*n* = 3).

Previous studies have shown that gadolinium element exerts cytotoxic activity against isolated mitochondria [24,36]. In this regard, we studied NP-induced mitochondrial dysfunction by determining the production of reactive oxygen species using the MitoSox Red probe. A short 5-h treatment with NaGdF_4_ NPs was sufficient to increase staining intensity with the MitoSox Red probe, indicating a rise in superoxide production (Figure 5C). Interestingly, we did not observe elevated levels of other reactive oxygen species (such as hydroxyl radical and peroxynitrite anion), as determined by staining with the H_2_DCFDA probe (Figure S4, Supplementary Data), which was observed in other studies [25]. The increase in superoxide concentration was further accompanied by more intense staining with the MitoTracker DeepRed mitochondrial probe, indicating mitochondrial biogenesis after an 18-h exposure to NPs (Figure 5D). Since the MitoTracker probe does not discriminate well between defective and intact mitochondria [37], this increase in the mitochondrial compartment size, along with superoxide production, may suggest both the proliferation of healthy mitochondria [38] and the accumulation of defective ones. We examined NP-induced changes of in the mitochondrial membrane potential using the JC-1 fluorescent probe to test this hypothesis. Treatment with NaGdF_4_-based NPs resulted in a concentration-dependent and significant decrease of red fluorescence, indicating the impairment of mitochondrial homeostasis, which confirmed the presence of defective mitochondria (Figure 6A). Damaged organelles, including mitochondria, are often subjected to cellular recycling through macroautophagy. Interestingly, 24-h treatment with NaGdF_4_ NPs increased the expression of LC3B-II, an autophagy marker and protein implicated in autophagosome formation (Figure 6B). This result suggests the presence of autophagic response to mitochondrial damage induced by NaGdF_4_-based NPs. Again, it should be emphasized that despite some variation in the rate of response (i.e., the rate was the fastest for S1 and slowest for S3), all these observations were made for treatments with NPs of all available sizes.

### 3.4. Disruption of Mitochondrial Homeostasis Induces Apoptotic Cell Death

**Figure 7:**
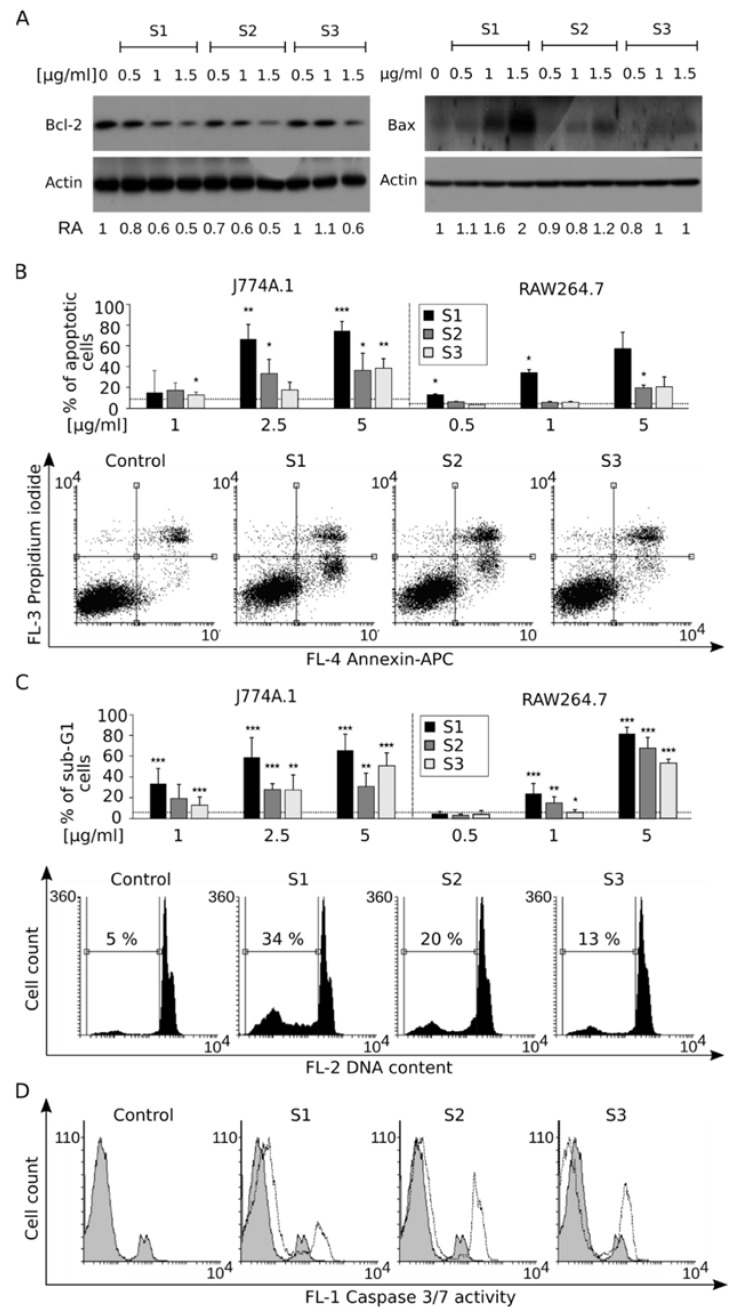
(**A**) Western blots of pro-apoptotic Bax and antiapoptotic Bcl-2 after a 24-h treatment with NPs (J774A.1). Bax and Bcl-2 protein expressions normalized relative to β-Actin (RA). Representative blots are shown; (**B**) Dot-plot analysis of Annexin V and propidium iodide staining indicating the apoptotic process. Representative dot-plots are shown. Bar graphs show the analysis of three independent experiments. The averages of apoptotic cells (AnnexinV+/PI− and AnnexinV+/PI+) are shown on the bar graph along with SD; (**C**) Propidium iodide staining of apoptotic cells. The marked population represents cells with subdiploidal DNA content indicating the final phase of apoptotic process. Representative histograms are shown. Bar graphs show the analysis of 3 independent experiments ± SD. * *p* < 0.05; ** *p* < 0.01; *** *p* < 0.001 All comparisons were performed with respect to the control; (**D**) Activation of caspases 3 and 7 of the execution phase in J774A.1 cells. Cytofluorometric analysis using a peptide cleaved by activated caspases 3 and 7. Representative histograms are shown (*n* = 3).

The mitochondrial membrane functions as a rheostat deciding about cell death and survival under stress conditions. In simplified terms, this can be described as a balance between anti-apoptotic and pro-apoptotic proteins. We found that a 24-h treatment with NaGdF_4_ NPs led to downregulation of the anti-apoptotic Bcl-2 protein and upregulation of a proapoptotic Bcl-2 family member—Bax (Figure 7A). Both changes may be direct triggers, leading to the execution of apoptosis. The externalization of phosphatidylserine (Figure 7B), accompanied by the impairment of cell membrane integrity (i.e., demonstrated by propidium iodide incorporation) upon NaGdF_4_ treatment, are indicators of the ongoing cell death process. Activation of an execution phase protease, caspase-3 and 7, confirms the ongoing apoptosis in cells treated with NaGdF_4_ NPs (Figure 7C). Genome fragmentation is the final stage of apoptotic cell death. We found that all NPs induced DNA fragmentation despite some variation in the extent of the process (Figure 7D). Similarly as in the previous section, the induction of cell death-related phenomena could be observed after treatment with NaGdF_4_ NPs of all sizes.

### 3.5. Exogenous Soluble Gadolinium or Fluoride Did Not Decrease the Viability of RAW264.7 Macrophages

**Figure 8:**
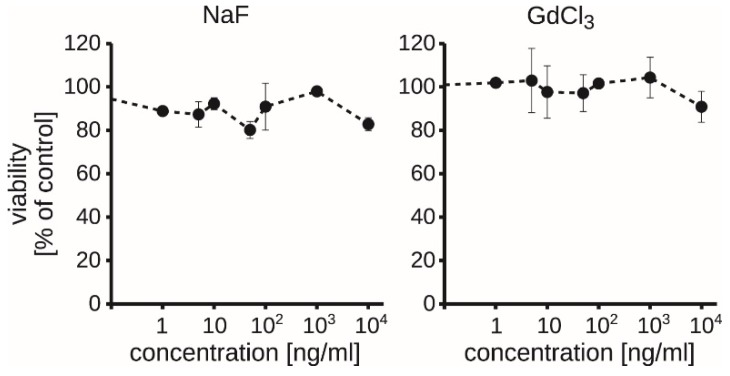
Viability of RAW264.7 cells ± SD after a 48-h treatment with soluble gadolinium and fluoride salts (*n* = 3).

As some authors observed the Gd^3+^-related toxicity [39,40] and we can’t exclude dissolving of NPs in cell culture media or an ion leakage, we asked whether the presence of Gd^3+^ or F^−^ in cell culture media was sufficient to reproduce the toxicity of NaGdF_4_ NPs. To address this question, the cells were treated with gadolinium and fluoride salts in a wide range of concentrations corresponding to concentrations relevant to ion leakage from NPs. We found that neither Gd^3+^ itself nor F^−^ induced cytotoxic effect when present in cell culture media (Figure 8). Accordingly, we did not observe apoptosis induced by gadolinium (III) chloride or sodium fluoride (not shown). This indicates that gadolinium exerts the cytotoxic effect as a solid nanomaterial formulation, and toxicity cannot be merely attributed to Gd^3+^ cation or fluoride anion present in cell culture media.

### 3.6. Inhibition of Cell Acidification Prevents Cytotoxicity of NaGdF_4_ Nanoparticles

**Figure 9:**
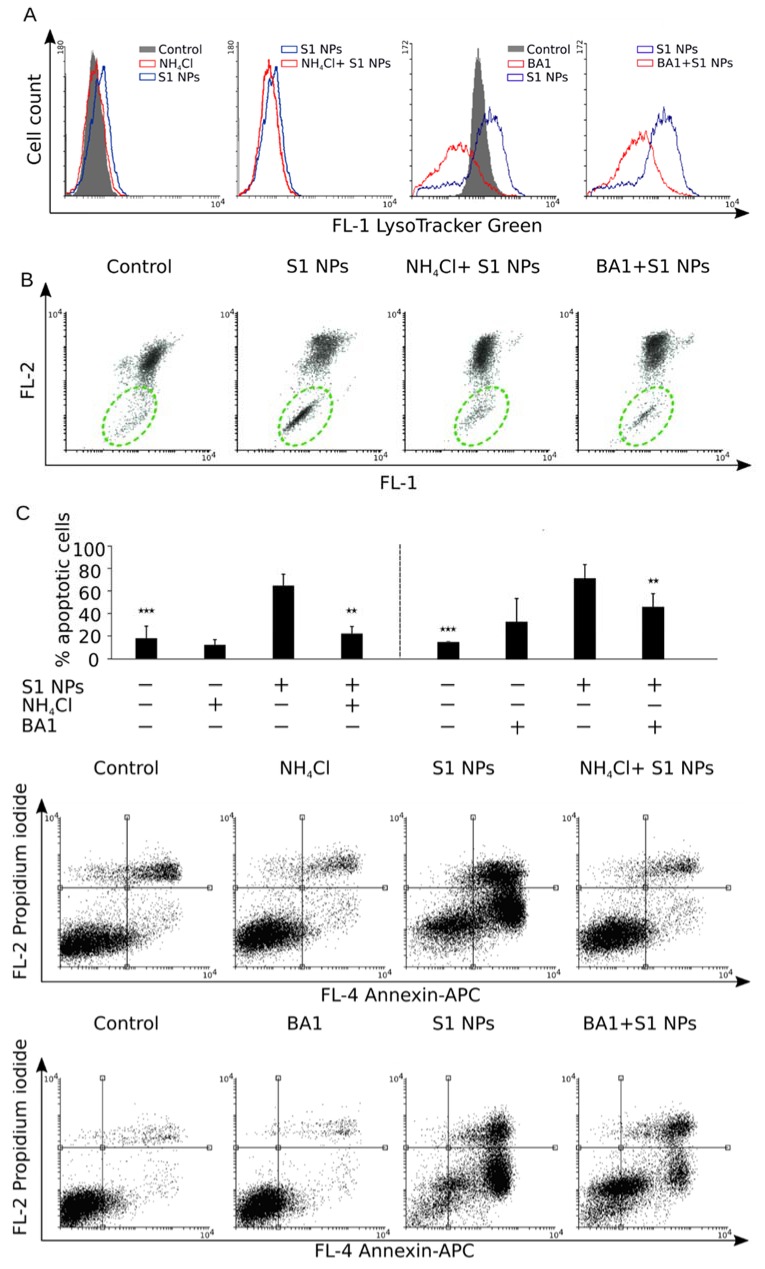
The effect of acidification inhibition of the cells on apoptosis induced by NaGdF_4_-based nanoparticles (2.5 µg/mL). (**A**) 50 nM Bafilomycin A1 (BA1) or 5 mM NH_4_Cl prevents acidification detected by Lysotracker green; (**B**) dot-plot analysis of the mitochondrial potential (MMP) measured using the JC-1 mitochondrial probe. Representative dot plots are shown; (**C**) Dot plot analysis of Annexin V and propidium iodide staining indicating the apoptotic process. Representative dot-plots are shown. Bar graphs show the analysis of 3 independent experiments. The average of apoptotic cells are shown (AnnexinV+/PI− and AnnexinV+/PI+) ± SD. * *p* < 0.05; ** *p* < 0.01; *** *p* < 0.001. All comparisons were performed with respect to S1 NPs treated cells.

Lysosomal targeting of NPs was proposed as important element toxicity mechanisms of various nanoparticles, including NaGdF_4_ NPs [21,22]. Similarly, lysosomal targeting of NaGdF_4_-based NPs is at the heart of the mechanism observed in our studies harsh and acidic conditions present in the activated macrophage phagolysosome may be crucial for the destabilization of the integrity of gadolinium fluoride NPs within the cells. This can lead to intracellular gadolinium and fluoride ion leakage and its mitochondrial toxicity. In view of negligible or absent cytotoxicity of gadolinium (III) chloride or sodium fluoride in the medium, it is important to say that these are NPs that mediate the transfer of Gd^3+^ and F^−^ ions (in the form of crystalline NP) to the cell interior, leading to the consequent cytotoxicity induction. For this purpose, we verified whether the inhibition of cell acidification would diminish NPs cytotoxicity. We used two well-known acidification inhibitors (i.e., Bafilomycin A1 and ammonium chloride) to verify whether its blockage was able to rescue the cells from NP- induced apoptosis. Firstly, ammonium chloride or Bafilomycin A1 had a minimal impact on the cells and did not interfere with S1 NPs internalization (Figure S5, Supplementary Data). Secondly, as expected, a 2-h pre-treatment of J774A.1 cells with these compounds prevented the increase of the lysosomal compartment and intracellular pH, as assayed by LysoTracker Green (Figure 9A). Moreover, pre-treatment with Bafilomycin A1 or ammonium chloride prevented the disruption of mitochondrial potential (Figure 9B) and further execution of apoptosis, which could be observed as a significant decrease in the number of apoptotic cells upon treatment with S1 NaGdF_4_-based NPs (Figure 9C). This clearly indicates that cell acidification is crucial for the cytotoxic effect of NaGdF_4_ NPs and strongly suggests that cytotoxicity is caused by intracellular destabilization of nanoparticles, although it should be noted, based on pH stability studies, that low pH conditions of the lysosome seem to be insufficient to destabilize NaGdF_4_-based NPs [21,22].

## 4. Discussion

In the present study, we demonstrated toxic effects of low doses of NaGdF_4_ nanoparticles on activated macrophages. Interestingly, we found that other cell lines, both cancer and normal, seemed to be fairly resistant to treatment with gadolinium-based nanoparticles, similarly as in most of the previous studies (reviewed by [18]). This indicates that the high phagocytic activity of RAW264.7 and J774A.1 cells is important, but not sufficient (data not shown) for the cytotoxic effect to occur. To date, the toxic effect of gadolinium-based nanoparticles has been demonstrated in several studies performed on normal or cancer cells, but the concentrations used in the studies ranged from 10 to 100 µg/mL [25,26,41,42]. In contrast, in our work, the IC_50_ of S1 NPs in RAW264.7 and J774A.1 was 0.81 ± 0.06 µg/mL and 0.52 ± 0.04 µg/mL, respectively. We observed fast interaction/uptake of NaGdF_4_-based NPs by these cells. It is interesting to inquire to what extent the high efficacy of gadolinium-based contrast agents designed for tumor imaging comes from their preferential uptake by tumor-associated macrophages, which are a substantial part of the growing tumor mass [14,43,44].

In the present study, we have used NaGdF_4_ nanoparticles with intentionally different sizes, but similar surface chemistry and ξ-potential. It should be noted that the toxic effect (with a similar underlying mechanism) was observed regardless of the nanoparticles size or shape. The cells use different strategies to absorb particles of different sizes. For example, phagocytosis is used by specialized cells like macrophages, and allows to ingest large particles, while clatrin-dependent mechanism of endocytosis dominates for smaller nanoparticles [45,46]. Our data suggest that cytotoxicity does not discriminate between small and large NPs. Some differences in the magnitude of the effect or its rate were observed, but most probably they could be attributed to the geometry of NPs or due to difficulties in comparing the doses of insoluble nanomaterials of different sizes and shapes that can could depend on the actual number of NPs, its quantity (mass) and area.

The toxicity of gadolinium is very often discussed beyond its formulation and data obtained using Gd^3+^ ions are extrapolated to unique nanomaterials. Studies showing detrimental effect of gadolinium ions on isolated mitochondria neglect the whole aspect of Gd^3+^ internalization and interaction with cellular machinery. Here, we showed, that despite the high sensitivity to NaGdF_4_-based NPs, RAW264.7 cells had a very high tolerance to Gd^3+^ and F^−^ ions applied extracellularly in the form of soluble salts; concentrations as high as 10 µg/mL did not result in a significant viability loss. On the other hand, the particular composition (doping) of NaGdF_4_ had only a minimal effect on cytotoxicity. It clearly indicates that NaGdF_4_ at the cellular level should not be regarded merely as the source of particular ions, but also as a vector facilitating their very efficient and rapid uptake to certain cells. It is consistent with a relatively high tolerance to gadolinium and limited toxicity of gadolinium-based MRI contrast, which can originate from the limited penetration of Gd^3+^ ions into cells [47].

Based on the obtained results, we propose that the toxic reaction against the activated macrophages involves targeting nanoparticles to lysosomes, as previously proposed [48,49]. We have observed in all cases an increase of the lysosomal compartment upon treatment with NaGdF_4_-based nanoparticles, and it was visible as fast as 5 h after treatment. Additionally, we showed that pretreatment of macrophages with different cell acidification inhibitors (basic salt—ammonium chloride or proton pump inhibitor—Bafilomycin A1) led not only to the abrogation of lysosomal compartment growth, but more importantly, it prevented the loss of mitochondrial potential and apoptosis induced by NaGdF_4_. Therefore, we identified acidification of the lysosomes as a key event in the development of toxic reaction. This seems to contradict the high stability of NaGdF_4_ NPs at low pH, but we should appreciate the fact that the environment of the activated macrophage lysosome is significantly more severe than in any other cell, and involves the production of volatile oxygen species [23]. Thus, not only low pH, but also the presence of volatile reactive oxygen species seems to be needed to destabilize the structure of NaGdF_4_ crystals, thereby leading to NP etching and intracellular release of free Gd^3+^ ions from the crystalline particles. This has been previously shown to have a detrimental effect on mitochondria [24,36]. In this regard, we have observed a late, strong, anti-mitochondrial effect of NaGdF_4_ NPs accompanied by the generation of superoxide and further decrease of the mitochondrial potential. It is worth emphasizing that this effect was induced by a small quantity of NPs and it was observed in the whole cell system.

At the same time, NaGdF_4_ treatment led to the enlargement of the mitochondrial compartment (MitoTracker) and increased expression of the autophagy-related form of LC3B-II. This is consistent with and can be attributed to the increased autophagy of mitochondria damaged by Gd^3+^. This stress response aimed for cell rescue, often induced by NPs [26,49,50,51], seems to be an insufficient protection mechanism. We showed that the expression of the proapoptotic Bax was increased, while anti-apoptotic Bcl-2 reduced after 24 h of treatment. This event in itself could serve as a trigger of the apoptotic process [52], which we observed at many different levels. Firstly, we found that an early apoptotic event of cellular membrane destabilization was correlated with energetic crisis (Annexin V staining); next, activation of the execution phase caspases 3 and 7, and finally characteristic features of apoptotic cell death and genome fragmentation were observed.

The results from different time points emphasize the dynamic nature of toxic reactions occurring in the cell and support the proposed toxicity mechanism. It can be divided into several phases: first, the uptake, which in selected cell lines is very rapid and efficient; second, the storage and destabilization, represented by an increase of the lysosomal compartment and superoxide induction, followed by events directly leading to the induction of cell death, such as mitochondrial potential disruption, shifting balance of Bcl-2 family proteins and apoptosis-related events like DNA fragmentation, caspase 3 and 7 activation, phosphatydylserine externalization, loss of cell membrane integrity and low cell count (decreased viability of cell culture).

It seems that the toxic reaction is limited to the nanoparticle form of gadolinium and professional phagocytes, because this cell model enables nonspecific entry into the cells and only (?) professional phagocytes to destabilize NaGdF_4_ nanocrystals. This hypothesis is supported by the fact the cytotoxic effect was studies the same NPs in other cellular models. Moreover, such high of toxic properties activated macrophages can be easily overlooked in vivo, as the detrimental health effect can be observed only in infected or preconditioned animals, which is a rare case in proper animal handling. Additionally, our results also suggest that enhancing chemical stability or applying intentionally designed coatings with agents resistant to harsh conditions present in the phagolysosomes of activated macrophages should effectively prevent cytotoxicity in these cells.

## 5. Conclusions

In the present study, we have shown the cytotoxic effect of NaGdF_4_ nanoparticles against activated RAW264.7 and J774A.1 macrophages with a low IC_50_ of 0.81 µg/mL and 0.52 µg/mL, respectively. We provided a time frame for the cytotoxic process, which involved rapid internalization (1 h), increase of the lysosomal compartment and generation of superoxide (5 h), disruption of mitochondrial homeostasis (18 h) and induction of apoptosis (48 h), as a result of shifting the balance of Bcl-2 family proteins (24 h). Additionally, despite the stability of NPs at low pH, we recorded cell acidification (lysosomes) as a key event for cell death induction. Inhibition of acidification *almost completely* prevented the apoptosis induced by NaGdF_4_ nanoparticles. On the other hand, no other cell lines were sensitive to a low concentration of NaGdF_4_ nanoparticles. Therefore, chemical stability of lanthanide NPs in severe conditions of activated macrophage phagolysosomes must be considered as a necessary condition for their biocompability and biosafety.

## Figures and Tables

**Table 1:** The toxicity of NaGdF_4_ lanthanide doped nanoparticles against the macrophage cell lines. The IC_50_ (µg/mL) (Promega MTS assay) values are shown (*n* = 5); n.a. not assessed.

	20 nm	20 nm	S1	S2	S3
	NaGdF_4_	NaGdF_4_:Eu	NaGdF_4_:Yb;Er	NaGdF_4_:Yb;Er	NaGdF_4_:Yb;Er
RAW264.7	n.a.	1.87 ± 0.07	0.81 ± 0.06	1.33 ± 0.07	1.58 ± 0.05
J774A.1	1.13 ± 0.09	n.a.	0.52 ± 0.04	1.81 ± 0.05	4.04 ± 0.20

## Supplementary Materials

The following are available online at http://www.mdpi.com/2218-273X/9/1/14/s1, Figure S1: Size distribution of NaGdF_4_-based NPs measured by TEM, Figure S2: SEM images of interactions of 5 μg/mL NaGdF_4_-based NPs with J774A.1 after a 1-h treatment, Figure S3: SEM images and energy dispersive X-ray (EDX) analysis of NaGdF_4_-based NPs (5 μg/mL) in J774A.1 cells after a 1-h incubation, Figure S4: Reactive oxygen species (ROS) induction after a 5- or 16-h incubation with NaGdF_4_-based NPs. Figure S5: SEM images of the interaction of 5 μg/mL NaGdF_4_-based NPs with J774A.1, Figure S6: Lack of detectable fluorescence, NaGdF_4_ dotted with Yb and Er (S2) in conditions used in the study.
