# Supplementary material for: Toxicity Mechanism of Low Doses of NaGdF4:Yb3+,Er3+ Upconverting Nanoparticles in Activated Macrophage Cell Lines
Source: Biomolecules. 2019 Jan 3;9(1):14. doi: 10.3390/biom9010014 (PMC6359000; doi:10.3390/biom9010014)
Supplement: Supplementary file 1 [file biomolecules-09-00014-s001.pdf]

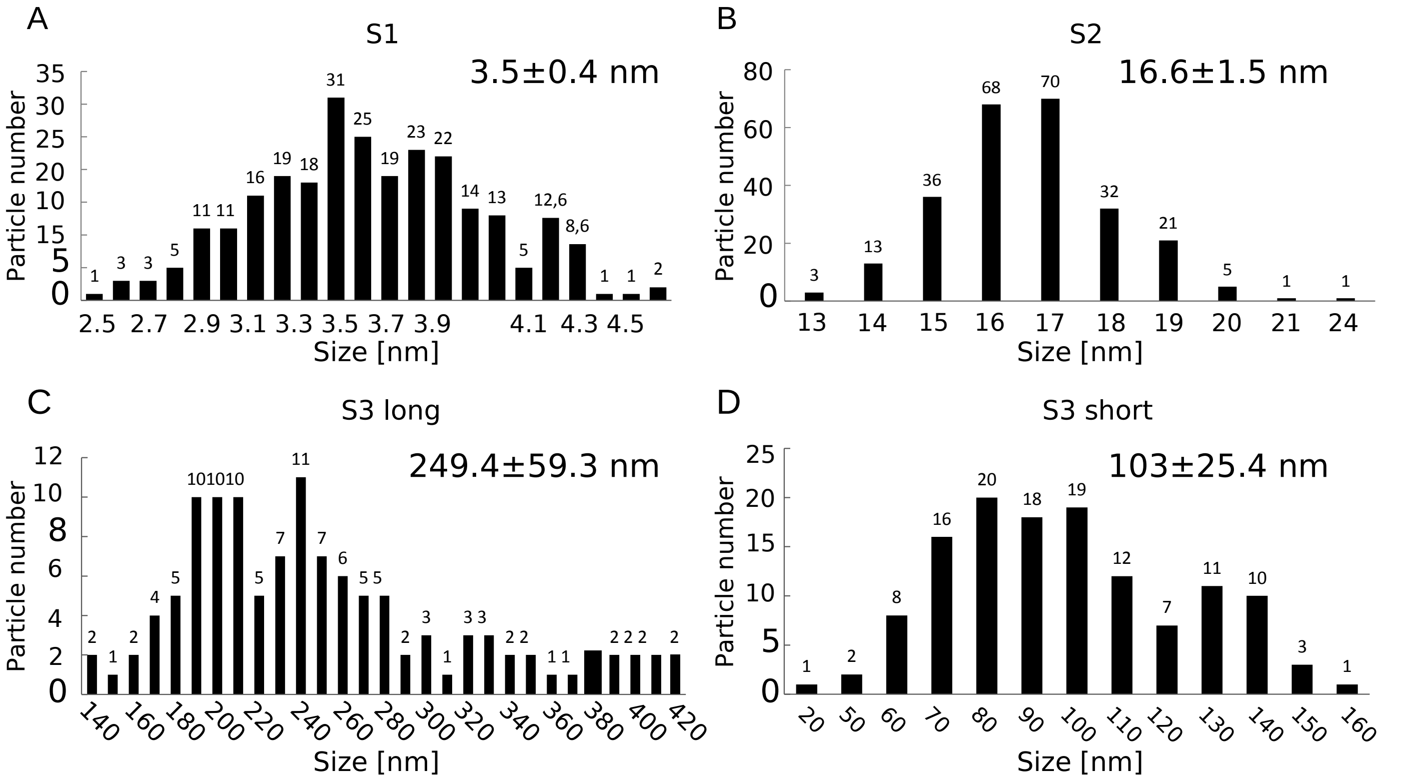

Figure S1. Size distribution of $NaGdF_4$ based NPs measured by TEM. A) S1 NPs B) S2 NPs C) The long side of S3 NPs D) The short side of S3 NPs. In the upper right corner the averages of dimeter with SD are shown.

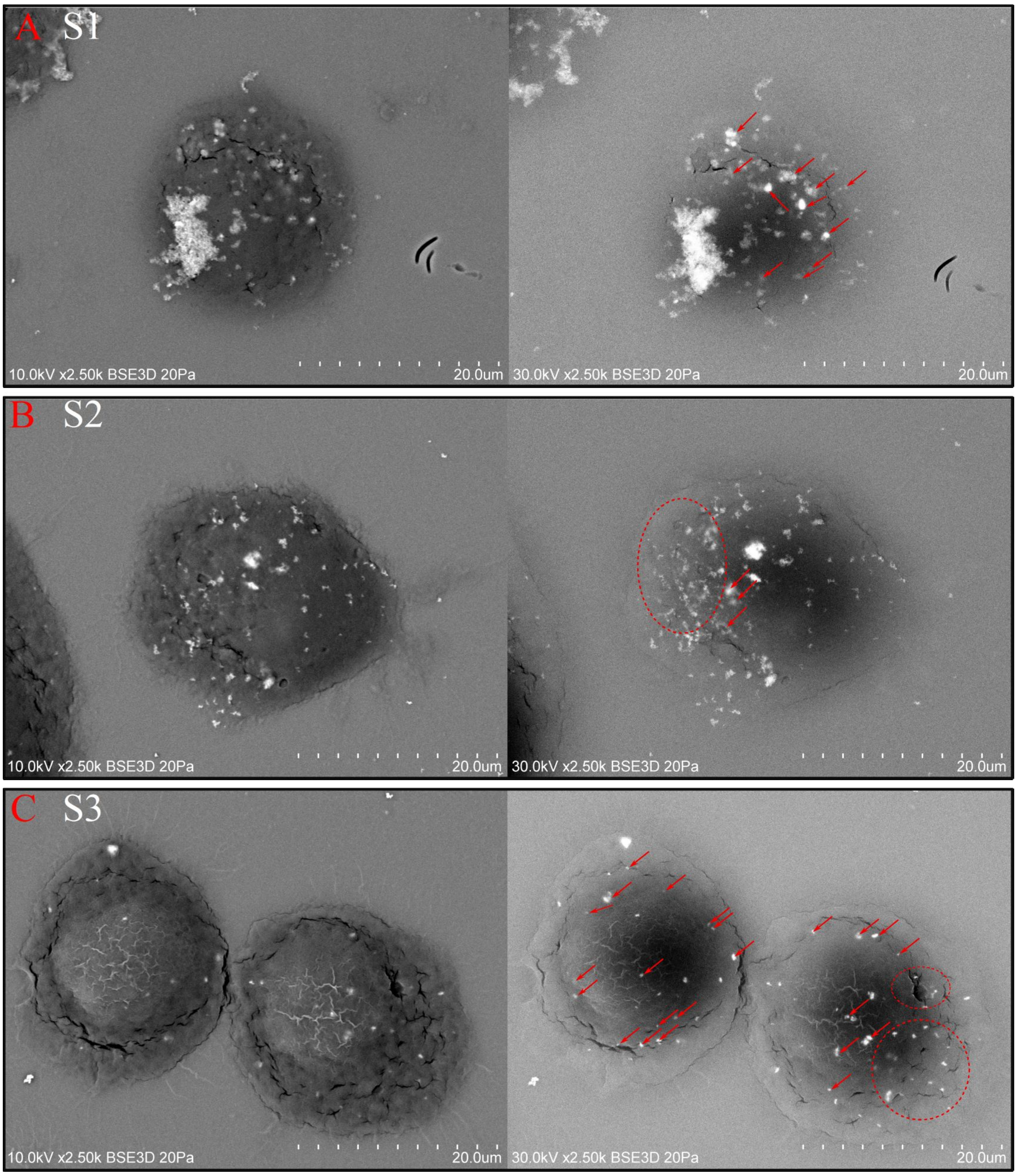

Figure S2. SEM images of interactions of 5 µg/ml NaGdF4 based NPs with J774A.1 after 1h treatment. (A) S1, (B) S2, (C) S3 NPs. The left side represents energy 10.0 kV and right side 30.0 kV (deeper penetration of the cell).

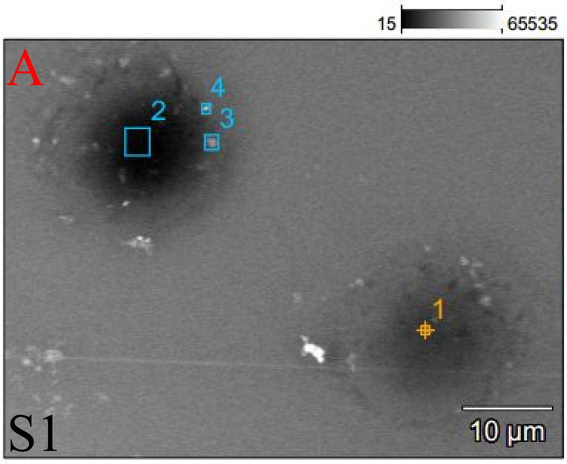

S1

Weight %

	C-K	O-K	Na-K	Mg-K	Al-K	Si-K	P-K	S-K	K-K	Ca-K	Gd-L
pt1	31.85	24.38	6.27	1.90	0.57	28.38	1.05	0.22	0.69	4.01	0.69
pt2	46.55	19.01	5.26	1.51	0.41	22.51	0.68	0.26	0.52	3.30	
pt3	20.65	35.12	8.42	2.19	0.58	28.17	0.38	0.08	0.62	3.61	0.17
pt4	18.18	36.74	8.44	2.34	0.58	28.56	0.41	0.08	0.66	3.58	0.41

Atom %

	C-K	O-K	Na-K	Mg-K	Al-K	Si-K	P-K	S-K	K-K	Ca-K	Gd-L
pt1	46.36	26.63	4.76	1.37	0.37	17.66	0.59	0.12	0.31	1.75	0.08
pt2	61.55	18.87	3.64	0.98	0.24	12.73	0.35	0.13	0.21	1.31	
pt3	31.16	39.79	6.64	1.64	0.39	18.18	0.22	0.05	0.29	1.63	0.02
pt4	27.84	42.24	6.75	1.77	0.39	18.71	0.25	0.05	0.31	1.64	0.05

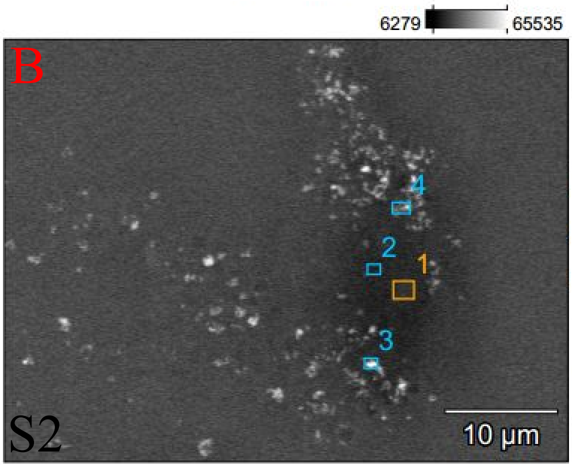

S2

Weight %

	C-K	N-K	O-K	F-K	Na-K	Mg-K	Al-K	Si-K	P-K	S-K	K-K	Ca-K	Co-K	Gd-L	Yb-L
pt1	23.64	0.00	30.83		7.66	2.22	0.63	29.62	0.36	0.10	0.74	4.19			
pt2	24.76	0.00	29.63		6.94	2.19	0.61	30.03	0.34	0.19	0.78	4.45	0.09		
pt3	22.01	0.00	27.09	3.41	7.35	2.21	0.59	30.57	0.23	0.12	0.70	4.26		1.14	0.32
pt4	14.65		35.33	2.50	7.88	2.50	0.58	30.83		0.12	0.68	4.24		0.54	0.15

Atom %

	C-K	N-K	O-K	F-K	Na-K	Mg-K	Al-K	Si-K	P-K	S-K	K-K	Ca-K	Co-K	Gd-L	Yb-L
pt1	35.55	0.00	34.81		6.02	1.65	0.42	19.05	0.21	0.06	0.34	1.89			
pt2	37.17	0.00	33.39		5.44	1.63	0.41	19.28	0.20	0.11	0.36	2.00	0.03		
pt3	34.12	0.00	31.53	3.34	5.96	1.69	0.41	20.27	0.14	0.07	0.33	1.98		0.13	0.03
pt4	23.20		42.02	2.51	6.52	1.96	0.41	20.89		0.07	0.33	2.01		0.07	0.02

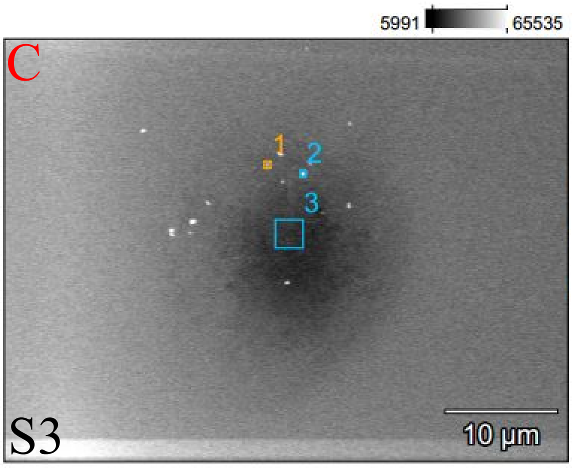

S3

Weight %

	C-K	O-K	F-K	Na-K	Mg-K	Al-K	Si-K	P-K	S-K	K-K	Ca-K	Co-K	Gd-L
pt1	7.89	43.46	1.03	8.83	2.51	0.72	30.56		0.15	0.65	3.74	0.18	0.28
pt2	7.60	43.78	1.21	8.63	2.54	0.68	30.50		0.16	0.67	3.62	0.11	0.49
pt3	28.35	29.10		8.08	2.10	0.59	27.04	0.37	0.19	0.62	3.45	0.12	

Atom %

	C-K	O-K	F-K	Na-K	Mg-K	Al-K	Si-K	P-K	S-K	K-K	Ca-K	Co-K	Gd-L
pt1	12.76	52.75	1.05	7.46	2.01	0.52	21.13		0.09	0.32	1.81	0.06	0.04
pt2	12.31	53.22	1.24	7.30	2.03	0.49	21.12		0.09	0.33	1.76	0.04	0.06
pt3	41.25	31.78		6.14	1.51	0.38	16.82	0.21	0.10	0.28	1.50	0.04	

Figure S3. SEM images and energy dispersive X-ray (EDX) analysis of NaGdF4 based NPs (5 µg/ml) in J774A.1 cells after 1h incubation. A) S1 B) S2 C) S3.

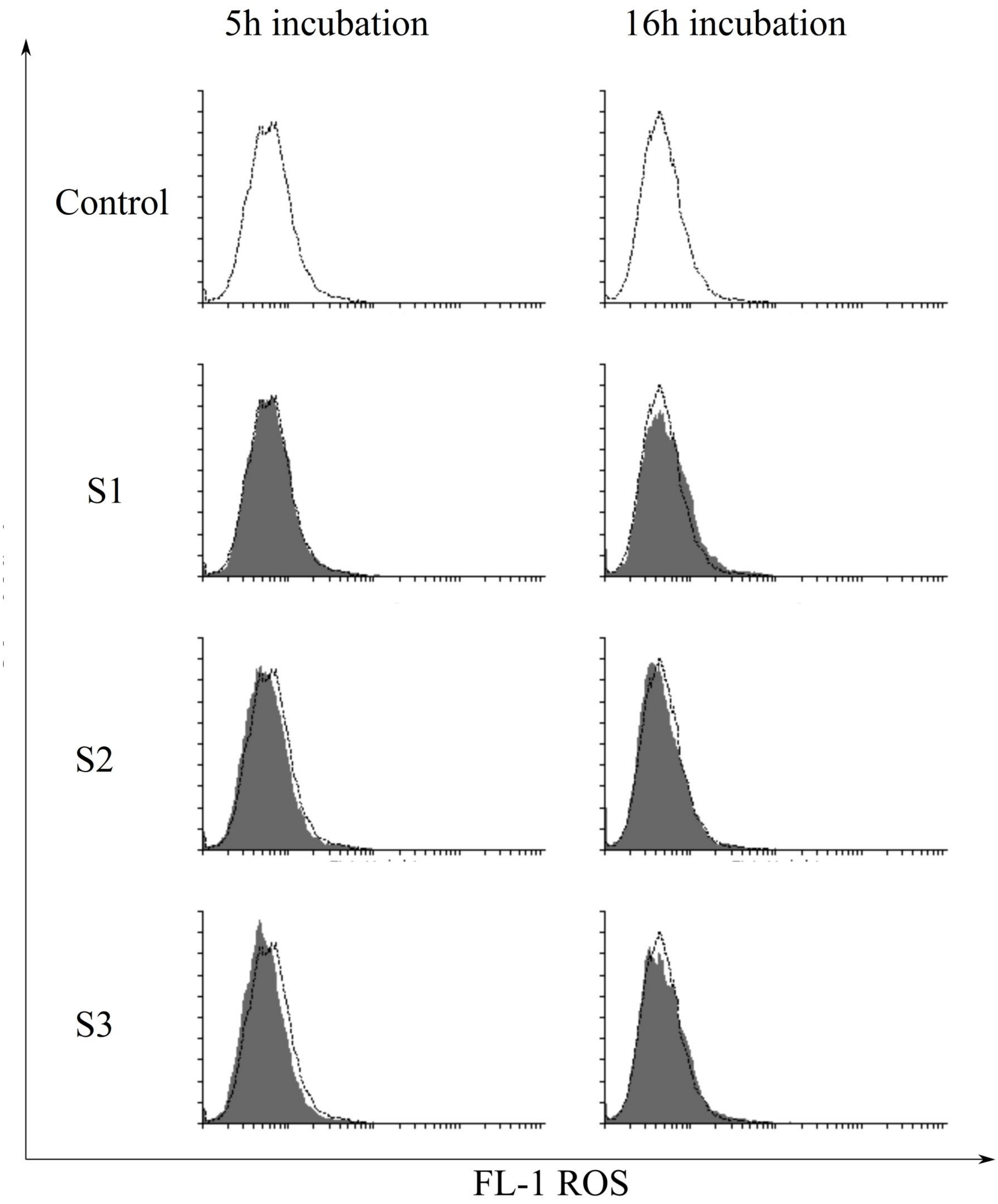

Figure S4. Reactive oxygen species (ROS) induction after 5 or 16h incubation with $NaGdF_4$ based NPs. Detection of ROS was performed using H2DCFDA dye. Shift into the right indicates rise of ROS in the cells.

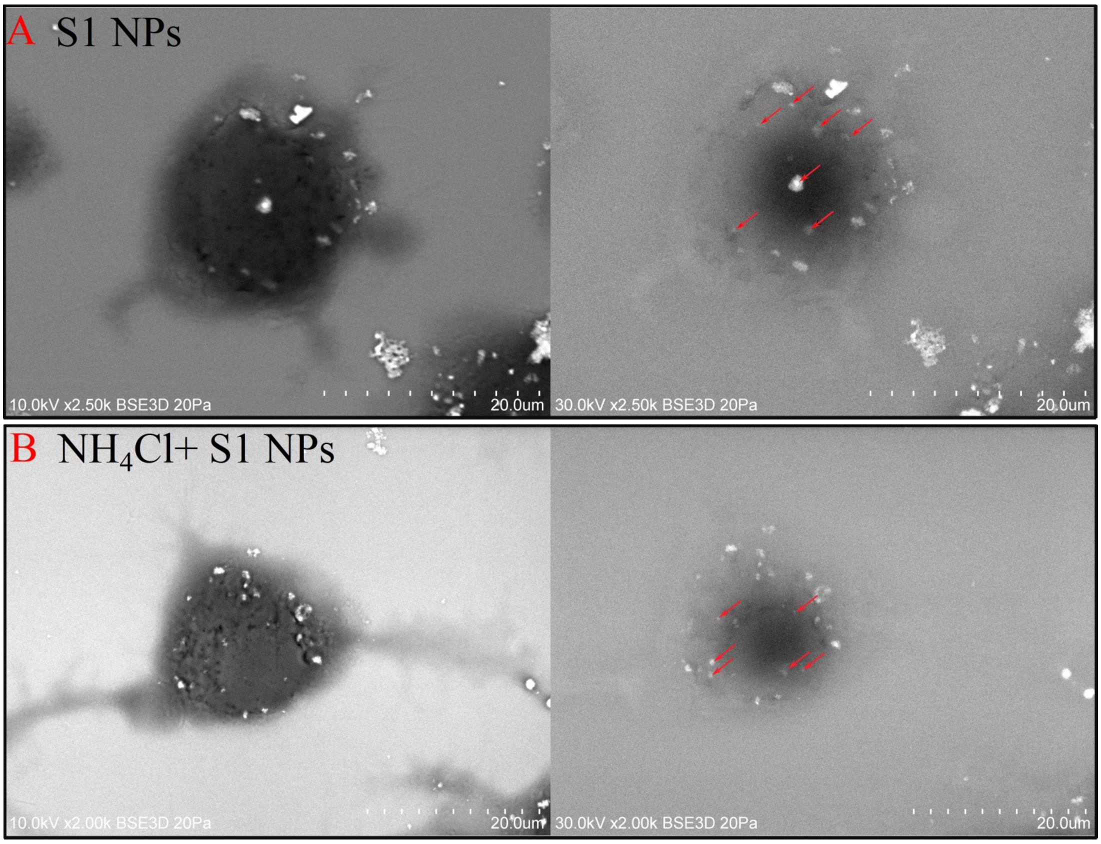

Figure S5. SEM images of interaction of 5 µg/ml NaGdF4 based NPs with J774A.1. A) Interaction after 1h incubation with S1 NPs, B) Interaction after 2 h preincubation with 5 µM NH_4Cl and then 1h with S1 NPs.

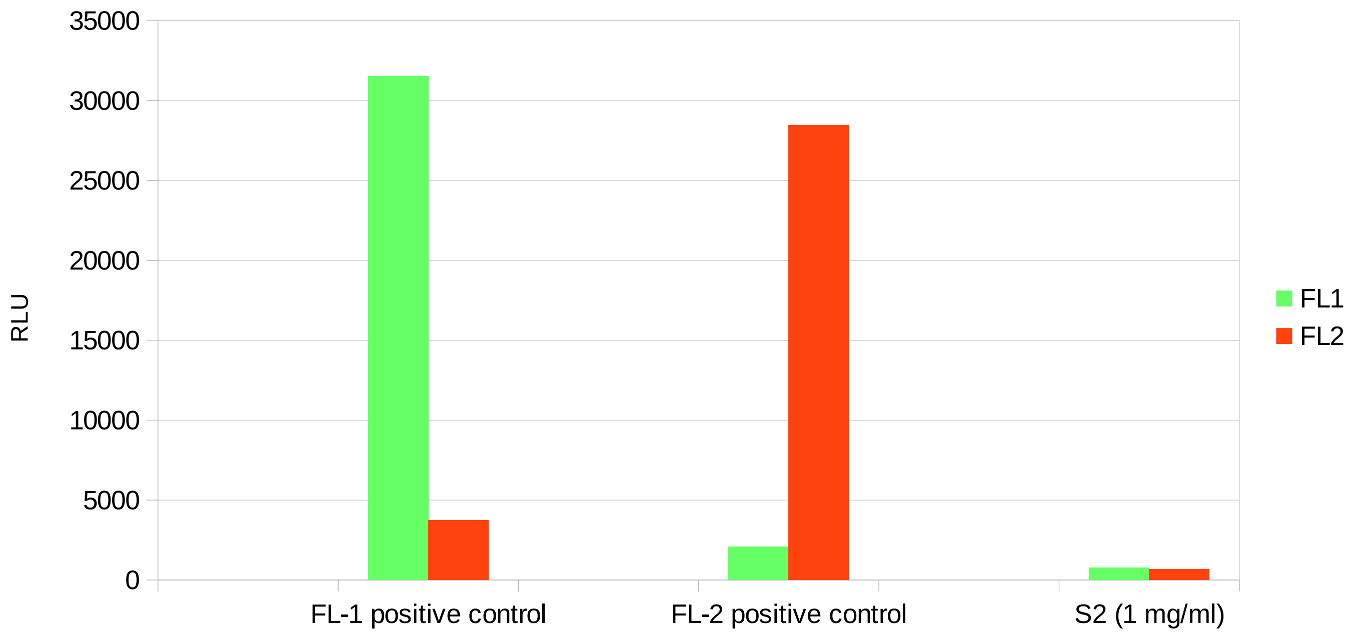

Figure S6. Lack of detectable fluorescence NaGdF4 dotted with Yb and Er (S2) in conditions used in the study.
